# CONSEQUENCES OF PERCEIVED STRESS ON COGNITIVE DECLINE AND BLOOD PRESSURE AS A MODERATOR IN OLDER BLACK AMERICANS

**DOI:** 10.1093/geroni/igad104.3091

**Published:** 2023-12-21

**Authors:** DeAnnah Byrd, David Coon, Roland J Thorpe, Keith Whitfield

**Affiliations:** Arizona State University, Phoenix, Arizona, United States; Arizona State University, Phoenix, Arizona, United States; Johns Hopkins University, Baltimore, Maryland, United States; University of Nevada, Las Vegas, Las Vegas, Nevada, United States

## Abstract

Previous research suggests that perceptions of stress shape cognitive health outcomes. However, few studies have explored this association longitudinally. The aim of this study was to determine whether perceived stress is associated with cognitive changes over time and if blood pressure (BP) acts as a moderator in a sample of older Blacks 48 to 95 years of age, who were enrolled in the Baltimore Study of Black Aging—Patterns of Cognitive Aging and interviewed at two time points, approximately 3 years apart (N = 450). Cognition included 5 domains: working memory, processing speed, verbal memory, vocabulary, and inductive reasoning. Three readings of orthostatic BP were collected and mean systolic (range = 82-230.3 mmHg) and diastolic BP (range = 51-136.7 mmHg) values were calculated for each participant. Results from the linear regression model showed no direct effect of perceived stress on cognitive decline. Systolic (F(3,426)= 4.37, p= 0.0048) and diastolic (F(3,426) = 3.26 p=0.0215) BP modified the relationship between stress and inductive reasoning at follow-up but was not related to any other cognitive domains, adjusting for age, sex, education, depressive symptoms, comorbidities, and baseline cognition. These important findings demonstrate the joint effects of stress and BP on cognition, highlighting the need for more research documenting the underlying processes and risk factors for domain-specific cognitive decline. The potential benefits of this approach provide a basis for developing cognitive interventions for this population of Black Americans.

